# Tracking modern human population history from linguistic and cranial phenotype

**DOI:** 10.1038/srep36645

**Published:** 2016-11-11

**Authors:** Hugo Reyes-Centeno, Katerina Harvati, Gerhard Jäger

**Affiliations:** 1 Paleoanthropology, Senckenberg Center for Human Evolution and Paleoenvironment, Eberhard Karls University of Tübingen, Rümelinstraße 23, D-72070 Tübingen, Germany; 2 DFG Center for Advanced Studies ‘‘Words, Bones, Genes, Tools’’, Eberhard Karls University of Tübingen, Rümelinstraße 23, D-72070 Tübingen, Germany; 3 Department of Linguistics, Eberhard Karls University of Tübingen, Wilhelmstraße 19, D-72074 Tübingen, Germany

## Abstract

Languages and genes arguably follow parallel evolutionary trajectories, descending from a common source and subsequently differentiating. However, although common ancestry is established within language families, it remains controversial whether language preserves a deep historical signal. To address this question, we evaluate the association between linguistic and geographic distances across 265 language families, as well as between linguistic, geographic, and cranial distances among eleven populations from Africa, Asia, and Australia. We take advantage of differential population history signals reflected by human cranial anatomy, where temporal bone shape reliably tracks deep population history and neutral genetic changes, while facial shape is more strongly associated with recent environmental effects. We show that linguistic distances are strongly geographically patterned, even within widely dispersed groups. However, they are correlated predominantly with facial, rather than temporal bone, morphology, suggesting that variation in vocabulary likely tracks relatively recent events and possibly population contact.

Early explorations on the association between languages and genes indicated that patterns of linguistic diversity paralleled those of genetic diversity. Most studies used pairwise distance measures of genetic and linguistic dissimilarity between populations in order to statistically compare the significance of their association^1^,^2^,^3^,^4^,^5^,^6^. Other work on the phylogenetic structure of genetic and linguistic data assessed similarities in the topology of generated trees^7^,^8^. In all, the general conclusion was that as modern human populations separated and became genetically differentiated, their languages followed a similar evolutionary trajectory. The call for a ‘new synthesis’^9^,^10^ was promptly issued, envisioning linguistic, genetic, and archaeological lines of evidence that would provide a coherent reconstruction of the human past. Contemporaneously, human palaeontologists and geneticists advanced the hypothesis that extant modern human populations stem from a common ancestral population that inhabited Africa approximately 100–200 thousand years ago (~100–200 ka)^11^,^12^. Whereas the pioneering studies on the gene-language association tested hypotheses concerning the origins and dispersal of European peoples and languages within a historical time period^1^,^2^,^3^,^5^,^13^,^14^, subsequent worldwide studies attempted to find an association into a pre-historical time depth. As with genes, it was hypothesized that languages spoken by extant African populations could hold traces of the ancestral ‘mother tongue,’ with Khoisan click languages (consisting of phonemes characterized by obstruent consonants) being the best candidates^15^,^16^. Drawing from Darwin’s idea of constructing a phylogeny of languages^17^, the reasoning of this hypothesis was that if the evolutionary principle of common descent and modification could be applied to genes, then so, too, could it be drawn for languages. Here, we aim to revisit the question of how languages and inherited biological traits are associated by taking advantage of the fact that skeletal components of modern human crania differentially correlate with neutral genetic polymorphisms. We seek to assess the association of cranial shape and language in order to understand to what extent language can be used to reconstruct population history.

For much of recent human evolution, genetic drift—changes that are due to stochastic rather than directed processes—is considered to be the primary mode by which hominin populations became differentiated^18^,^19^. In modern humans, genetic diversity within populations has been found to decrease with increasing distances from Africa—a pattern that is attributed to a serial increase in genetic drift following an expansion out of the place of origin^20^. This pattern has also been observed for skeletal phenotype data^21^,^22^,^23^,^24^,^25^ and, intriguingly, for phonemic language data^26^. While the pattern between geography and phonemes is supported only under strict assumptions of phonemic inventories and accumulation rates^27^, the observed loss of phonemic diversity has nevertheless suggested to some that language traits can be used to reconstruct deep population relationships. By this logic, the temporal depth of reconstruction would be at least as far back as the genetic divergence of Khoisan-speaking populations, ~40 ka^28^,^29^, and possibly into the time of the common ancestral population^26^. However, most linguists view this possibility with great caution, in general favouring a more shallow, historical time depth due to the difficulty in distinguishing between common descent and other mechanisms, such as linguistic convergence, chance resemblances, word borrowing, and other non-stochastic processes^30^.

Indeed, while genetic drift may be one of the primary evolutionary modes of differentiation in modern human populations, gene flow is an important factor that can act to reduce diversity among populations. Gene flow between populations increases their similarity via the exchange and mating of individuals. Similarly, active communication between speakers of different languages can lead to borrowing, ultimately making these languages appear more similar to each other. As such, it is not only the diversity within populations that must be examined, but also the diversity between populations. In addition to the negative correlation between intra-population diversity and increasing distances from Africa, a serial founder model also predicts a positive association between inter-population differences (biological distance) and geographical separation (geographical distance)^20^. Geographical distance is one of the main factors limiting gene flow, as populations close to each other are more likely to meet and exchange genes in comparison to populations far from each other. Furthermore, land-based geographical distances are more highly correlated with genetic distances—a result that considers oceans as barriers of movement and that is attributed to a model of the primary modes of dispersal from the African birthplace and into other parts of the world^20^. A positive, statistically significant relationship between land-based geographical distances and biological distances is consistently observed for genetic and skeletal data^20^,^31^, but not for phonemic data^32^,^33^. Such a relationship is also expected among languages and dialects from the same language family (i.e. a group of languages whose common descent has been demonstrated conclusively by historical linguists). A recent study showing significant correlation between geographic distances and linguistic distances, partially within and partially across language family categories^34^, relied on Ruhlen’s controversial classification of linguistic phyla^15^ rather than on raw variables of linguistic characteristics, such as lexical similarity or grammatical structure. The question of whether vocabulary lists contain historical information beyond the limits of established language families, therefore, remains controversial.

Over the last decade, important progress has independently been made in both skeletal shape analysis and historical linguistics. In the former case, advances have allowed for better quantification of variation between populations and species, proving useful in the assessment of phylogenetic affinities of previously contentious taxonomic categories (e.g. refs 35 and 36). Importantly, with the use of these methods, consensus has emerged on the differential preservation of population history in modern human cranial shape^36^,^37^,^38^,^39^,^40^,^41^. Whereas the temporal bone has consistently shown a significant correlation with neutral genetic markers, the facial region of the cranium shows a weaker correlation and is instead more strongly associated with environmental variables. Moreover, the temporal bone is thought to reflect population affinities at a deep temporal scale while the neurocranium and face reflect more recent associations between populations^36^,^37^,^41^. Thus, from a theoretical standpoint, cranial phenotypic data offer a unique way of calibrating to what extent language can track population history. Indeed, language can be considered an ‘extended phenotype’^42^, which, like the skeleton, is under influence of non-heritable factors or otherwise not directly regulated by the genotype.

In historical linguistics, phylogenetic methods from computational biology are now widely used in testing competing models of the prehistoric origins and spread of languages within a language family^43^,^44^,^45^,^46^. Comparisons of vocabulary lists across languages also suggest that deep historical signals can be detected^47^. These approaches are highly labour intensive, requiring expert classification, and currently available for only a handful of language families. However, using an exceptionally large database of vocabulary data covering about two thirds of extant worldwide languages^48^, it has recently been shown that automated phylogenetic inference based on phonetic distances between words is in excellent agreement with expert classification^49^,^50^. This weighted alignment method may therefore allow for an accurate reconstruction of population history.

To test the hypothesis that vocabulary information can be used for reconstructing population history at a deep temporal scale, we first quantified the association of geographical distances (*G*) with vocabulary distances. We defined a purely data-based vocabulary distance measure (*L*) between languages. From this we derived an aggregate linguistic distance measure between language families (*F*), defined as the arithmetic mean of the pairwise distances between their component languages. The aggregate geographic distance *G* between two families was likewise calculated as the arithmetic mean of the corresponding distance between the component populations. This allowed us to compare the variation across *N* = 265 language families and isolates. Since genetic and skeletal phenotypic distances between populations are significantly correlated with land-based *G*, a significant association between *F* and *G* would imply a linguistic spatial patterning consistent with a serial founder model.

Secondly, we sampled eleven populations from Africa, Asia, and Australia (Table S1) in order to compute the distances between their languages (*L*) and, in turn, evaluate the association of *L* with *G* and with biological distances. For *L*, we used the weighted alignment method using vocabulary data^49^. For biological distance, we calculated phenotypic distances (*P*_*ST*_) using cranial shape data. The *P*_*ST*_ measure is analogous to the genetic distance measure *F*_*ST*_, with an underlying assumption that the phenotype reflects the net effect of polygenic inheritance^51^,^52^,^53^. Following refs 36, 37 and 41, we partitioned our phenotype dataset into three regions: the temporal bone, the face, and the neurocranium. Since the temporal bone has been shown to correlate at higher degree to neutral genetic variation, *P*_*ST*_ values derived from it should correlate with *L* to a greater degree than with other cranial regions if vocabulary distances indeed parallel neutral genetic distances through a deep temporal scale. We statistically assessed the associations between *P*_*ST*_, *L*, and *G* using Mantel and Dow-Cheverud correlation tests.

## Results

The association between *G* and *F* was significant (*r* = 0.276, *p* < 0.0001); thus, geography explains ~8% of variance between language families. In the association between *L* and *P*_*ST*_, the whole cranium had the highest correlation and, independently, all of its regions showed statistical significance (Table 1). Of the three cranial regions, the highest correlation with language was for the face, followed respectively by the neurocranium and the temporal bone. In order to assess the extent by which linguistic and cranial diversity was patterned by geography, we computed the correlation of *L* or *P*_*ST*_ with *G*. We found a significant correlation in all cases, with the highest values for the whole cranium and face configuration (Table 2). Geography explains up to half of overall cranial shape variation and when considering its component parts, it explains respectively ~42%, ~15%, and ~20% of variation in facial, neurocranial, and temporal bone phenotype variation. It also explains ~16% of lexical variation in languages.

In order to factor out the effect that geography has on linguistic and phenotypic variation, we computed partial correlations between phenotypic and linguistic distances, conditioned on land-based geographical distances (Table 3). In this case, the face configuration had the highest correlation with *L*—one that was higher than for the whole cranium. Of the three cranial regions, only the face configuration was below the Bonferroni correction threshold for multiple model comparisons. In the sequential Bonferroni correction, the neurocranium configuration was also significantly associated with language. The Dow-Cheverud tests show that facial shape is more strongly correlated to linguistic distances than the temporal bone, even after controlling for geographic distance (Table 4).

## Discussion

The significant association we found between *G* and *F* is comparable to that reported by ref. 34. These results further validate the automated weighted alignment method for generating linguistic distances. The positive association between geography and the two thirds of extant world languages represented by our dataset is consistent with the predictions of a serial founder model and, more generally, with a spatial patterning of vocabulary. Furthermore, for our eleven sampled populations, the correlation between *G* and *L* was also significant. Because we found that the spatial patterning applied to the cranial phenotype data as well, it was necessary to consider this as a confounding variable in the association between language and cranial phenotype. In other words, any relationships observed between *L* and *P*_*ST*_ could potentially be explained by the fact that both are correlated with *G*. In partial correlations of *L* and *P*_*ST*_ while controlling for *G*, the facial configuration had the highest correlation. These are surprising results since a strong correlation between *L* and temporal bone *P*_*ST*_ is expected if vocabulary and genetic diversity follow a parallel evolutionary trajectory that is primarily consequent of common descent. Because we found that, instead, facial shape was more strongly correlated with language, it is necessary to consider other mechanisms that could have generated the observed pattern of vocabulary diversity present in today’s languages.

Our results, which derive from vocabulary data spanning three continents, suggest that finding clear gene-language associations at a substantially greater spatial and temporal time depth may be elusive. Previously, vocabulary data from well-studied language families, including Indo-European^43^,^44^ and Austronesian^45^,^46^, have been used for testing competing language dispersal scenarios, spanning a time depth into the early Holocene, ~9 ka. These results are largely in agreement with archaeological and genetic lines of evidence for population dispersals. Comparisons of vocabulary lists across Eurasian languages have more recently attempted to extend this limit to the Palaeolithic, ~14.5 ka^47^. It has previously been suggested that the temporal bone reflects population history since the divergence of African and Eurasian populations^36^,^41^,^54^. Since we do not find a strong association between *L* and temporal bone *P*_*ST*_ after controlling for geography, our results do not support the use of vocabulary to effectively reconstruct the human past as far back as the last common ancestor in Africa, as previously hypothesized^26^. Nevertheless, *L*’s spatial patterning, as well as its association with aspects of cranial phenotype, suggests that vocabulary data retain a certain level of information regarding recent population history. Future work, particularly with advancements in dating techniques using linguistic data, may provide a better estimate for the temporal limits of vocabulary as a tool for reconstructing population history.

Early studies on the association of cranial regions and neutral genetic markers suggested that such differential correlations could reflect differences in skeletal development^36^,^37^. For example, whereas the basicranium develops early in life, with some components (e.g. the petrous pyramid of the temporal bone) almost fully formed in utero, other regions form later in life and are subject to epigenetic effects. Therefore, it was hypothesized that certain components of the cranium, which develop early in life and at a relatively fast rate, would evolve slowly while those that develop later are less constrained and can evolve faster. This evolutionary-developmental hypothesis has been partly tested recently with work showing that the temporal bone of the cranium has a significant correlation with neutral genetic markers, beginning at an early ontogenetic stage^40^. Temporal bone shape is also more associated to neutral genomic variation in comparison to the whole cranium when controlling for population divergence time and considering different population sizes^41^. Differences in the evolutionary rate of change are also relevant for language since most linguists consider vocabulary to change in a highly dynamic manner^30^. Within the framework of the evolutionary-developmental hypothesis and in the absence of selection, the language-face association might therefore be most parsimoniously attributed to faster rates of change.

The rate of change of vocabularies is estimated to be between 3–4 times faster in comparison to changes in their grammar^55^. Although grammar data are not currently available for the populations we sampled, recent studies have found a strong association between genes and grammar data for populations within Europe^56^ and across Europe, Africa, and Western Asia^57^. It has previously been hypothesized^34^,^58^ that grammatical rate of change is more comparable to the rate of change of some genetic systems than to that of others, which has been partly supported for a sample of populations from Eurasia and Africa^57^. We therefore hypothesize that the strong association of lexical variation and facial shape variation might reflect a correspondence in evolutionary rates of change. To further explore this hypothesis, data comprising of (*i*) both lexical and grammatical variables, (*ii*) distinct skeletal phenotypic variables, and (*iii*) various genomic polymorphisms for the same populations would be ideal. Likewise, further empirical work and simulation approaches that vary the rate of anatomical and lexical characters under diverse evolutionary models will serve to validate or falsify this hypothesis.

While not mutually exclusive, other interpretations can be offered for our results. First, the association we found between *L* and face *P*_*ST*_ might be broadly attributed to epigenetic effects on phenotype. For example, by extension of the known association between environmental variables and facial phenotype^36^,^37^,^59^, it is possible that the significant association between lexical and facial shape variation in our results is due to how both are associated to structured environmental variation. Second, a more complex, adaptive interpretation may entail mechanisms akin to natural or sexual selection acting on both facial shape and vocabulary. Finally, a third and more parsimonious hypothesis is that vocabulary and facial shape both reflect recent admixture between populations. Indeed, in explaining the biological variation of extant populations, an alternative to the serial founder effect model has recently been proposed^60^, emphasizing natural selection and admixture with few or no bottlenecks. Testing for explicit environmental correlates and selective pressures, as well as understanding skeletal phenotype and language change after population admixture, will be necessary for addressing these different possibilities.

We caution that interpretation of our results is bound to the limitations of our dataset and study design. In particular, landmark configurations in our study capture diverse anatomical characters to varying degrees. For example, while the landmark configurations for the face and temporal bone both comprise thirteen landmarks each, the neurocranium only comprises eight. More generally, the face and temporal bone configurations capture more anatomical details than the neurocranial configuration. Thus, the best comparison is between the face and the temporal bone, which indeed results in a statistically significantly different association with language. An additional limitation to our study is that we did not consider differences in population size or divergence time, which can be informative in understanding the effect of drift experienced by populations and which can serve to contextualize pairwise *P*_*ST*_ values^41^,^54^. We note that, from a linguistic perspective, genomic estimates of effective population size could also inform estimates of speaker population size, which have traditionally been limited to recent census data but which are important in modelling linguistic evolution. Therefore, a possible avenue of future work is to formulate a quantitative genetic approach in measures of linguistic diversity and linguistic distances, which has, to our knowledge, not been applied to language datasets.

Having already made substantial progress since the modern evolutionary synthesis^61^ of the mid-twentieth century, the new synthesis at the end of the twentieth century aimed to unify the understanding of the diversification of languages, cultures, and peoples. Incorporating the humanities in understanding the history of populations seemed an essential component of human evolutionary biology. Our study adds to the goals of the new synthesis by emphasizing the ability to incorporate a line of evidence—skeletal phenotype—shaped by both heritable and non-heritable factors. It thus serves to calibrate the associations observed between genes and languages alone. Our study also outlines productive areas of future research within this research program. New avenues of research now provide further ways to test multidisciplinary approaches in addressing questions of the human past.

## Methods

### Cranial Phenotype data

Our data collection procedure follows refs 36 and 37. Sampled crania are from the Holocene modern human ethnographic and archaeological collections housed at the Musée de l’Homme, National Museum of Natural History (Paris, France). Crania were selected on the basis of adult ontogeny and the absence of bone pathology, balancing population samples by sex to the extent possible, for a total of *N* = 265 (Table S1). For each specimen, a total of thirty-two anatomical landmarks—in the form of 3D coordinates—were collected by H.R.-C. using a MicroScribe G2X desktop digitizer. Landmark measurement error was tested by digitizing a specimen ten times across the span of a week. Error ranged from 0.183–2.175 millimetres (mm) or 0.147–4.892%. In the few cases where cranial preservation precluded data collection, missing landmarks were estimated by reflected relabelling of the bilateral homologue^62^ using the Morpheus software^63^. Specimens with missing data along the midline were not included. A generalized Procrustes analysis (GPA) was used to superimpose the raw coordinate data using the MorphoJ v1.05 software^64^. Following GPA, four datasets were generated: one that included all data (32 landmarks), and three that separately represented the neurocranial (8 landmarks), facial (13 landmarks), and temporal bone (13 landmarks) segments of the cranium. Separating the dataset after GPA has the effect of considering the location of each segment relative to the others, ensuring the retention of positional information. We performed a principal component analysis (PCA) in order to determine which PC scores to use for calculating *P*_*ST*_. Currently, no consensus exists on the number of PC variables that should be included for arriving at population distances. Therefore, we chose a systematic, three-step ‘stopping rule’^65^ approach to statistically assess which PCs to include. First, we performed 10,000 bootstrap replicates on the shape variable data of each cranial configuration. Second, the bootstrapped components were re-ordered and reversed in order to increase correspondence with the original, empirical axes^66^. Third, we compared the 95% confidence interval of the bootstrapped eigenvalues with those expected under a random, hypothetical model^65^. At this point, our stopping rule was to include the PCs before the first point in which the 95% eigenvalue confidence interval was below the hypothetical trendline generated from the random model (Fig. S1). The PC selection procedure was carried out in the PAST v2.17b software^67^. We note that seven degrees of freedom are lost following Procrustes superimposition in three dimensions, accounting for scaling and for translation and rotation along each axis; therefore, the last 7 components are excluded by default. We also note that our PC selection method conforms to the common practice of excluding PCs that explain less than 1% of variance, as these components explain less of the variance than the original shape variables. Lastly, this PC selection approach is consistent with previous work showing that the number of PCs explaining a majority of variation is positively associated with the complexity of the cranial element, rather than the number of landmarks or total shape variables^39^,^68^. Respectively for the whole cranium, face, neurocranium, and temporal bone, the approximate amount of cumulative variance (i.e. eigenvalue percent) explained by the selected PCs was 77%, 70%, 88%, and 89% (Fig. S1).

Finally, we used the selected PCs to calculate *P*_*ST*_ using the RMET 5.0 software^69^. *P*_*ST*_ in this case is calculated following Harpending and Ward’s model^70^, where the mean of a quantitative trait is assumed to be proportional to the underlying mean allele frequency and where the variance is assumed to be proportional to heterozygosity. We assumed that all populations had proportionally equal demographic histories, i.e. population sizes. Because estimates of heritability differ and may be population specific^71^,^72^, we chose an approximation of heritability *h*^2^ = 0.3 in all calculations. We note that while population size and heritability estimates affect the magnitude of *P*_*ST*_ values by increasing or decreasing them, all pairwise *P*_*ST*_ changes are proportional and would therefore not affect subsequent matrix correlation analyses. Furthermore, while *F*_*ST*_ in population genetics ranges from 0–1—where 0 indicates no genetic differentiation (i.e. panmixia) and 1 indicates complete genetic differentiation—*P*_*ST*_ values contrast in that they can exceed the 1 threshold when using heritability and population size estimates. In spite of this, *F*_*ST*_ and *P*_*ST*_ are highly correlated under neutrality and in the absence of selection^51^,^52^,^54^ since the values between population pairs are proportional. Pairwise *P*_*ST*_ values for each cranial region are reported in Supplementary Tables S2 and S3.

### Language data

Linguistic distances (*L*) were computed from the Automated Similarity Judgment Program (ASJP) database^48^. ASJP is organized into doculects, which are coherently documented language varieties that may include different variants of the same language. For example, doculects sampled for our Japanese population included varieties of Japanese spoken in Tokyo as well as that spoken in Kyoto. ASJP is a collection of core vocabulary lists from over 6,000 doculects, covering about two third of the world’s extant languages. This database is confined to phonetic transcriptions and does not contain expert cognacy judgments. It has previously been shown that phylogenetic inference based on phonetic distances between ASJP word lists is in excellent agreement with expert classifications^49^,^50^. The ASJP word lists consist of words for 40 core concepts represented in each doculect (see Supplementary Information). They are verbalized in all modern human languages, express the same meaning across languages, are resistant to changes in meaning or to borrowing, and are largely independent of culture^73^. As such, distances derived from these can be considered neutral distances, making them comparable to distances derived from neutral genetics or their skeletal phenotypic correlates.

Distances between word lists were computed following the two-step procedure detailed in ref. 49. In the first step, similarity scores between individual words are determined using the Needleman-Wunsch algorithm^74^ and empirically estimated weights. For example, the English, German, and Spanish words for ‘hand’ (respectively, ‘hand’, ‘Hand’, and ‘mano’) are transcribed in the ASJP database as *hEnd*, *hant*, and *mano*. To estimate the similarity between the German word with the English and the Spanish word, the sound strings are pairwise aligned: *hEnd*-*hant* and *hEnd*-*mano*. This alignment corresponds to the Needleman-Wunsch algorithm, which is a standard bioinformatical method for aligning molecular sequences. Each pair of sounds is assigned a weight corresponding to the log-odds probability of the sounds being historically related versus the probability of being matched by chance^49^. In the German-English and German-Spanish example, both word pairs exhibit two matches and two mismatches. However, the mismatches (a-E and d-t) in the pair *hant-hEnd* reflect common sound changes while the mismatches (h-m and t-o) in *hant-mano* do not. This asymmetry is captured by the sum of the weights of the aligned sounds corresponding to the two alignments. For *hand-hEnd*, this sum is 4.80 while for *hand-mano* it is −11.85, so the former pair is a much better candidate for reflecting common descent than the latter. In the second step, the similarity between two doculects is quantified as the degree to which the distribution of string similarities between synonymous word pairs exceeds the distribution of string similarities between non-synonymous pairs. The distance between two doculects is defined as a linear function of the similarity with negative coefficient that has 0 and 1 as theoretical minimum and maximum, respectively.

Some ethnographic records were available for the cranial collections, but in most cases the languages spoken by the individuals could not be uniquely identified. ASJP contains meta-data for each doculect, such as geographic location and expert classifications. We chose a group of candidate doculects from the ASJP database for each population, using a combination of three heuristics. First, subpopulation information consisting of ethnic affiliation was used to narrow down the space of candidate languages. For instance, the South India population is specified as ‘Tamil’; hence Tamil is the only candidate doculect for it. Second, if the population was from islands (e.g. Japan, Melanesia, New Caledonia), only doculects from these islands were considered. Third, whenever no more specific information was available, the candidate doculects were those ASJP doculects whose geographic coordinates (according to the ASJP meta-data) are situated within a distance of at most 500 km from the population in question. The lists of candidate doculects for each population are given in the Supplementary Information. In all, linguistic distance (*L*) between population pairs was calculated as the arithmetic mean of all linguistic distances between candidate doculects assigned to each population (results reported in Table S4). Similarly, distances between two language families (*F*) were computed as the arithmetic mean of all linguistic distances between sampled doculets. We followed the classification of languages into families according to the World Atlas of Language Structure (WALS)^75^. This classification is fairly inclusive, assuming several large families such as Altaic or Australian.

### Geographic data

We computed land-based geodesic distances (*G*) between populations following the method from refs 20 and 26 (Table S5). In the latter, paths between locations on different continents were constrained to pass through key waypoints, namely Cairo (31 E, 30 N) linking Africa and Asia and Phnom Penh (105 E, 11.5 N) linking Asia and Australia. This approach considers oceans as barriers and, more broadly, represents a parsimonious model of human dispersal routes between continents. Calculations were made in the geopy Python package, which assumes a spherical terrestrial shape and a radius of ~6,373 km.

### Correlation tests

Statistical significance of the association between any two matrices was evaluated against a null distribution by the Mantel test procedure. Simple Mantel tests were used to assess the correlation between linguistic (*L* or *F*) and cranial phenotype (*P*_*ST*_) distances, as well as between these and geographical distances (*G*). We used partial Mantel tests to evaluate the association of *L* and *P*_*ST*_ when controlling for *G*. Correlations between distance matrices were computed as Spearman’s rank correlation coefficients, as the dependencies between linguistic distances and both cranial phenotype and geographical distances are non-linear. In order to assess whether the *P*_*ST*_ values of a given cranial segment were statistically correlated more with *L* than the *P*_*ST*_ values of another cranial segment, we applied the Dow-Cheverud test^76^. Because the Dow-Cheverud test has been shown to reject too often when data are spatially auto-correlated^77^, we also conducted it while controlling for *G*. In all cases, two tailed *p*-values were determined with 10,000 permutations. Likewise, Bonferroni corrections were applied to *p*-value results of the subset landmark configurations, including a correction for multiple model comparisons and a sequentially rejective correction^78^,^79^. The multiple model comparison was applied for comparison of the three landmark configurations subset from the entire cranium, i.e. the face, neurocranium, and temporal bone. Thus, for all comparisons, a Bonferroni correction for multiple model comparisons was set at 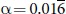 or α = 0.05/3, where 3 is equal to the number of subset landmark configurations being compared. By contrast, the sequential Bonferroni correction was set to a threshold of 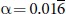 for the first lowest *p*-value value, then at α = 0.025 for the next lowest *p*-value value, and finally at α = 0.05. We applied these corrections to the Dow-Cheverud results but note that *p*-value significance thresholds in such a test have conventionally been set at α = 0.05 (e.g. refs 38,39,41 and 68). We further note that comparisons of each cranial subset landmark configuration against the whole cranium configuration is inadequate given the overlap of landmarks. We cross-checked Mantel results using the XLSTAT v2014.4.02 commercial software and the ecodist R package^80^, reporting here those derived from the former.

## Additional Information

**How to cite this article**: Reyes-Centeno, H. *et al*. Tracking modern human population history from linguistic and cranial phenotype. *Sci. Rep.* **6**, 36645; doi: 10.1038/srep36645 (2016).

**Publisher’s note:** Springer Nature remains neutral with regard to jurisdictional claims in published maps and institutional affiliations.

## Supplementary Material

Supplementary Information

## Figures and Tables

**Table 1:** Correlation of phenotypic and linguistic distances^1^.

Distance measure	Linguistic distance (L)
Whole Cranium *P*_*ST*_	*r* = 0.558; *p* < 0.0001
Face *P*_*ST*_	*r* = 0.545, *p* < 0.0001***
Neurocranium *P*_*ST*_	*r* = 0.483; *p* = 0.0003***
Temporal Bone *P*_*ST*_	*r* = 0.324; *p* = 0.013***

^1^Correlation *r* is Spearman coefficient value. Significance *p* value (two-tailed) is after 10,000 permutations. For the cranial subsets, ***significance after Bonferroni correction for multiple model tests, **significance after sequential Bonferroni correction, *significance at α = 0.05.

**Table 2:** Correlation of phenotypic or linguistic distances with geographical distances^1^.

Distance measure	Land-based *G*
Whole Cranium *P*_*ST*_	*r* = 0.721, *p* < 0.0001
Face *P*_*ST*_	*r* = 0.648, *p* < 0.0001***
Neurocranium *P*_*ST*_	*r* = 0.388, *p* = 0.004***
Temporal Bone *P*_*ST*_	*r* = 0.462, *p* = 0.0004***
Linguistic distance *L*	*r* = 0.402, *p* = 0.003

^1^Correlation *r* is Spearman coefficient value. Significance *p* value (two-tailed) is after 10,000 permutations. For the cranial subsets, ***significance after Bonferroni correction for multiple model tests, **significance after sequential Bonferroni correction, *significance at α = 0.05.

**Table 3:** Partial correlation of phenotypic and linguistic distances, controlling for geography^1^.

Distance measure	Linguistic distance (*L*)
Whole Cranium *P*_*ST*_	*r* = 0.370; *p* = 0.007
Face *P*_*ST*_	*r* = 0.395, *p* = 0.002***
Neurocranium *P*_*ST*_	*r* = 0.329; *p* = 0.017**
Temporal Bone *P*_*ST*_	*r* = 0.127; *p* = 0.357

^1^Correlation *r* is Spearman coefficient value. Significance *p* value (two-tailed) is after 10,000 permutations. Control for geography, *G*, is based land-based distances. For the cranial subsets, ***significance after Bonferroni correction for multiple model tests, **significance after sequential Bonferroni correction, *significance at α = 0.05.

**Table 4:** Dow-Cheverud tests^1^.

Cranial Region	Whole	Face	Neurocranium	Temporal
Whole		*r* = 0.187, p = 0.175	*r* = −0.016, *p* = 0.914	*r* = −0.325, p = 0.021
Face	*r* = −0.056, p = 0.675		*r* = −0.152, *p* = 0.255	*r* = −0.333, p = 0.015***
Neurocranium	*r* = 0.204, *p* = 0.110	*r* = 0.287, *p* = 0.028*		*r* = −0.255, p = 0.067
Temporal	*r* = 0.407, *p* = 0.005	*r* = 0.363, p = 0.009***	*r* = 0.196, *p* = 0.158	

^1^Below diagonal: values comparing the differential association of cranial landmark configurations pairwise (listed in the rows and columns) against language; above diagonal: values comparing the differential association of cranial landmark configurations pairwise against language, while controlling for geography. Positive *r* values (correlation coefficient ρ1Z) indicate that the cranial segment listed in the column is more strongly correlated with language than the cranial segment listed in the row and vice versa for negative *r* values. Significance *p* value (two-tailed) is after 10,000 permutations. For the cranial subset comparisons, ***significance after Bonferroni correction for multiple model tests, **significance after sequential Bonferroni correction, *significance at α = 0.05.
