# Supplementary material for: Tracking modern human population history from linguistic and cranial phenotype
Source: Sci Rep. 2016 Nov 11;6:36645. doi: 10.1038/srep36645 (PMC5105118; doi:10.1038/srep36645)
Supplement: Supplementary Information [file srep36645-s1.pdf]

Supplementary Information

Tracking modern human population history from linguistic and cranial phenotype
Hugo Reyes-Centeno, Katerina Harvati, Gerhard Jäger

Contents

Suppl. Figure S1. Scree plot PCA data from cranial shape variables 2
Suppl. Table S1. Population Samples 3
Suppl. Table S2. Cranial phenotype distances (P_{ST}): whole cranium & face 4
Suppl. Table S3. Cranial phenotype distances (P_{ST}): neurocranium & temporal bone 4
Suppl. Table S4. Linguistic distances (*L*) and Geographical distances (*G*) 4
Suppl. Note S1: Core vocabulary 5
Suppl. Note S2: Candidate doculects 5

Supplementary Figure S1. Scree plot PCA data from cranial shape variables.

Results for (a) whole cranium, as well as (b) face, (c) neurocranium, and (d) temporal bone configurations. Blue-line is empirical trend of eigenvalue percentages (vertical axis) for each principal component (horizontal axis). Red line is hypothetical trend of eigenvalue percentages based on a random model. Black bars are 95% confidence interval of eigenvalue percentages after 10000 bootstrap replicates. The “stopping rule” applied in this study was to isolate the first instance when the 95% confidence interval fell below that expected under a random model (indicated by an arrows). All components before this were used in the calculation of P_{ST} (e.g. 16 PCs for the temporal bone configuration).

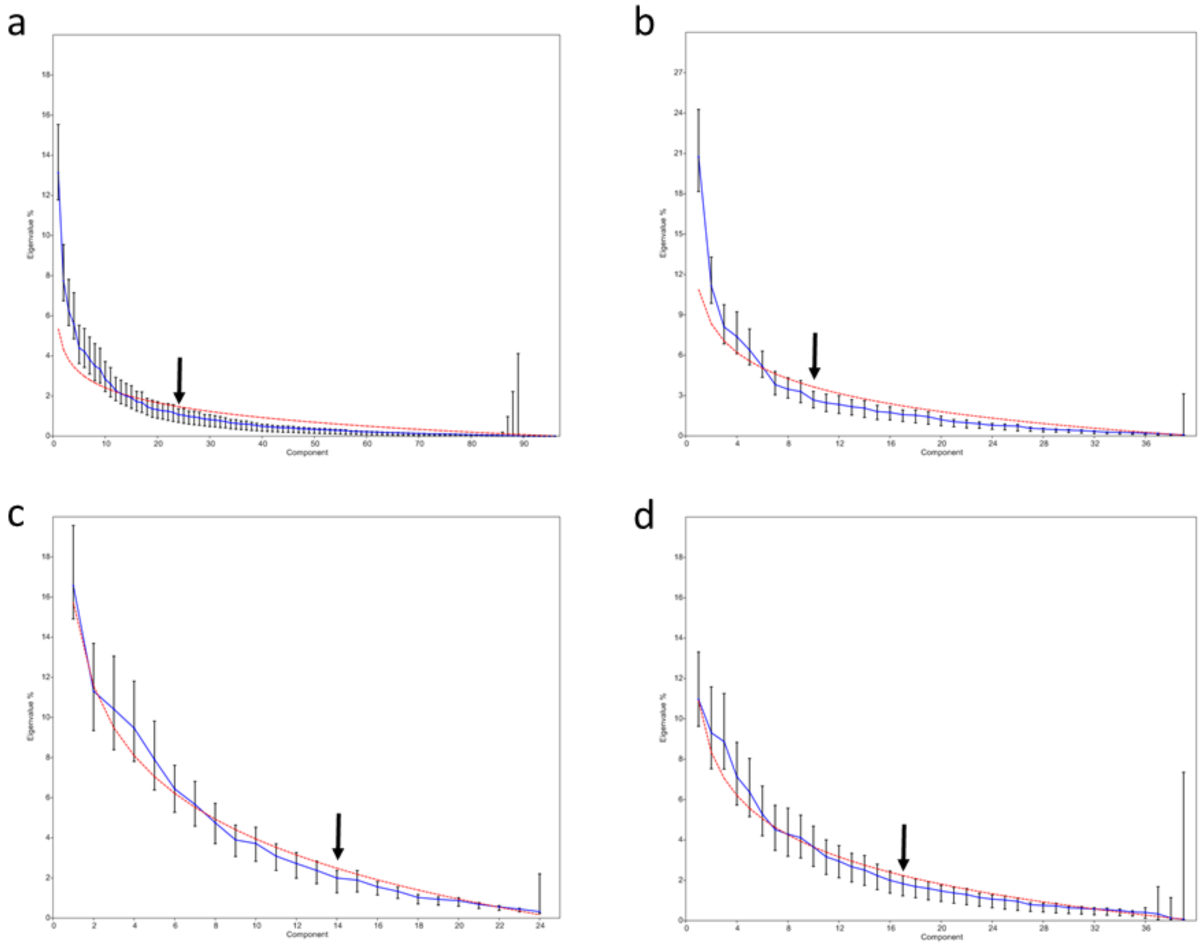

Supplementary Table S1. Population Samples

Populations		Subpopulations	Lat.	Long.	*N* Crania
AU	Australia	Australian	-33.89	151.24	26
CA	Central Asia	Dungan, Kalmyk, Tarantchi, Uyghur	43.29	68.26	30
EA	East Africa	Afar-Danakil, Amhara, Bouma, Glaba, Habesha, Igai, Karo, Koukou, Nyangatom, Pouma, Turkana	9.02	38.74	22
JP	Japan	Japanese	35.66	139.82	33
ME	Melanesia	Solomon & Vanuatu Islanders	-9.42	159.94	17
NC	New Caledonia	Bonde, Bouloupari, Gomen, Kanala, Ny, Tuauru	-22.28	166.46	26
NE	Philippines "Negrito"	Aeta, Agta	14.6	120.98	19
NG	Papua New Guinea	Papua New Guinea, Torres Strait Islanders	-9.48	147.19	23
NI	North India (Indo-European)	Bengali	28.63	77.2	15
SA	South Africa	Khoi, Malabar, Nama, San, Sotho, Tswana, Xhosa, Zulu	-26.2	28.05	20
SI	South India (Dravidian)	Maravar, Tamil	6.93	79.86	34

Supplementary Table S2. Cranial phenotype distances (P_{ST}): whole cranium & face[1]

Populations	AU	CA	EA	JP	ME	NC	NE	NG	NI	SA	SI
AU		2.613	1.268	2.259	0.398	0.441	1.067	0.566	0.827	1.120	0.543
CA	1.557		1.243	0.157	1.430	2.117	0.960	1.521	1.034	1.558	1.453
EA	1.045	0.986		1.442	1.024	1.431	1.085	1.167	0.900	0.054	1.011
JP	1.288	0.128	1.031		1.031	1.655	0.608	1.089	0.761	1.662	1.133
ME	0.380	1.046	0.893	0.749		0.146	0.572	0.105	0.269	0.961	0.174
NC	0.433	1.249	1.241	0.990	0.329		0.952	0.373	0.866	1.255	0.678
NE	0.743	0.903	1.054	0.723	0.588	0.777		0.318	0.528	1.154	0.743
NG	0.341	0.957	1.046	0.670	0.225	0.394	0.418		0.307	1.094	0.318
NI	0.471	0.628	0.740	0.494	0.414	0.675	0.560	0.354		0.850	0.034
SA	0.865	1.030	0.114	1.020	0.825	1.049	0.942	0.894	0.679		0.974
SI	0.480	0.884	0.951	0.726	0.464	0.559	0.752	0.474	0.045	0.858	

[1]Below diagonal: P_{ST} for whole cranium shape configuration, using 23 PCs, accounting for ~77% of variance (Fig. S1a). Above diagonal: P_{ST} for face shape configuration, using 9 PCs, accounting for ~70% of variance (Fig. S1b). Values rounded to the thousandths digit.

Supplementary Table S3. Cranial phenotype distances (P_{ST}): neurocranium & temporal bone[1]

Populations	AU	CA	EA	JP	ME	NC	NE	NG	NI	SA	SI
AU		0.572	0.523	0.512	0.300	0.420	0.543	0.044	0.279	0.342	0.440
CA	1.538		0.664	0.120	0.528	0.586	0.264	0.387	0.231	0.692	0.345
EA	0.676	0.627		0.528	0.397	0.615	0.886	0.550	0.379	0.146	0.633
JP	1.460	0.109	0.693		0.455	0.481	0.204	0.360	0.192	0.594	0.356
ME	0.387	0.951	0.462	0.741		0.229	0.682	0.254	0.231	0.342	0.217
NC	0.385	1.416	0.720	1.249	0.340		0.845	0.349	0.412	0.544	0.372
NE	1.047	0.754	0.566	0.526	0.551	0.999		0.417	0.346	0.706	0.531
NG	0.406	0.849	0.227	0.751	0.074	0.471	0.601		0.216	0.365	0.310
NI	0.507	0.428	0.504	0.409	0.351	0.592	0.587	0.339		0.300	0.048
SA	0.564	1.021	0.136	0.980	0.509	0.688	0.851	0.321	0.631		0.528
SI	0.551	0.737	0.731	0.686	0.397	0.426	0.848	0.480	0.081	0.750	

[1]Below diagonal: P_{ST} for neurocranium shape configuration, using 13 PCs, accounting for ~88% of variance (Fig. S1c). Above diagonal: P_{ST} for temporal bone shape configuration, using 22 PCs, accounting for ~89% of variance (Fig. S1d). Values rounded to the thousandths digit.

Supplementary Table S4. Linguistic (L) and geographical (G) distances [1]

	AU	CA	EA	JP	ME	NC	NE	NG	NI	SA	SI
AU		12019	17336	11436	2863	1970	6273	2746	10483	21127	9830
CA	0.86		6069	6013	11499	12674	6759	10210	1815	9859	4200
EA	0.858	0.868		12067	16816	17990	12076	15527	6920	4084	8168
JP	0.861	0.853	0.862		10916	12090	6176	9627	5851	15857	6862
ME	0.853	0.867	0.859	0.862		1590	5059	1398	9963	20607	9310
NC	0.858	0.868	0.863	0.866	0.784		6433	2500	11137	21781	10484
NE	0.86	0.866	0.876	0.844	0.779	0.803		3940	5222	15866	4570
NG	0.855	0.866	0.855	0.86	0.831	0.841	0.837		8674	19317	8021
NI	0.86	0.868	0.844	0.843	0.83	0.848	0.815	0.853		10711	2429
SA	0.86	0.86	0.861	0.855	0.86	0.868	0.865	0.859	0.866		11959
SI	0.859	0.854	0.862	0.874	0.865	0.844	0.877	0.858	0.848	0.865	

[1] Above diagonal: land-based distances geodesic distances rounded to the nearest km. Below diagonal: linguistic distance values rounded to the thousandths digit.

Supplementary Note S1: Core vocabulary

The ASJP dataset consists of core vocabulary found across two thirds of the world's extant languages. The 40-word list is as follows (in standard English language): ***I, you, we, one, two, person, fish, dog, louse, tree, leaf, skin, blood, bone, horn, ear, eye, nose, tooth, tongue, knee, hand, breast, liver, drink, see, hear, die, come, sun, star, water, stone, fire, path, mountain, night, full, new, name***.

Supplementary Note S2: Candidate doculects

Classification of doculects according to language family follows the WALS classification (ref. 75 of main text).

1. **Population**: Australia (AU)

   - **Language family:** Australian

   - **Candidate doculects**: (241 doculects)

     Ingura, Pungupungu, Wadjiginy, Wadyginy, Bunaba, Gooniyandi, Burarra, Gorogone, Djauan, Kitja, Miriwung, Kamor, Matngala, Yunggor, Gaagudju, Garawa, Erre, Mangerr, Urningangg, Buan, Gunbalang-Warlang, Gunwinggu-Manyallalukmayali, Gunwinggu-Gun-Djeihmi, Gunwinggu-Kune, Gunwinggu-Kunwinjku, Gunwinggu-Kuninjku, Kunkarakany, Amurdak, Iwaidja, Margu, Mawng, Djamindjung, Laragiya, Larrakia, Limilngan, Mangarayi, Alawa, Mara, Wandarang, Murrinh-Patha, Murrinhi-Patha, Djeebbana, Ngalakan, Ngandi, Mullukmulluk, Tyaraity, Nunggubuyu, Bardi, Yawuru, Adnyamathanha, Aghu-Tharrnggala, Akara, Alyawarr-Alyawara, Alyawarrmacdonalddowns-Alyawara, Alyawarra, Anmatyerre, Arabana, Aranda, Arrarnta-Western, Arrernte-Central, Arrernte-Eastern, Arrernteeastern-2, Awabakal, Badimaya, Banggarla, Barranbiya, Bayali, Bidjara, Bigambal, Birrdhawal, Birri-Australia, Bunganditj, Camberralanguage, Colac, Dalwongo, Darkinyung, Darling, Darrkinyung, Dhargari, Dhudhuroa, Dhurga, Dhuwal, Diyari, Djadjala, Djambarrpuyngu, Djapu, Djinang, Djinba, Duungidjawu, Dyaabugay, Dyangadi, Dyirbal, Gamilaraay, Gandangara, Gangulida, Gangulu, Gidabal, Gobabingo, Gomaidj, Gugu-Bujun, Gumbaynggir, Gunya, Gunyaguwamu, Gureng-Gureng, Gurindji, Guugu-Yimidhir, Guwamu-2, Guyambal, Jaru, Jiringayn, Kala-Laggaw-Ya, Kalkutung, Kanju, Karadjeri, Kaurna, Kaytetye, Kaytetye-2, Keramin, Kokata, Koko-Yalandji, Kuku-Uwanh, Kunggari, Kunjen, Kurrama, Kuuku-Yau, Lamalamacoastal, Lamalama-Inland, Linngithigh, Lower-Aranda, Madhi-Madhi, Madimadi, Malyangapa, Manggalili, Mantjiltjara, Mararba, Mar-gany, Martuthunira, Martu-Wangka, Mayaguduna, Mayi-Thakurti, Mayi-Yapi, Maykulan, Mbabaram, Mpakwithi-Anguthimri, Muruwari, Ngaanyatjarra, Ngadjunmaya, Ngalooma, Nganyaywana, Ngarigu, Ngawun, Ngunawal, Ngura, Nhanda, Nyangumarta, Nyawaygi, Nyungaeastern, Nyunga-Northern, Nyunga-South-Western, Omeo, Pallanganmiddang, Panytyima, Parimankutinma, Pintupi, Pitjantjatjarayankuntjatjara, Pitta-Pitta, Punthamara, Ritharngu, Southernaranda, Sydney, Thawa, Thayore, Thurawal, Umbuykamu, Uradhiangkamuthi, Uradhi-Atampaya, Uradhi-Yadhaykenu, Waalubal, Wa-gaya, Wajarri, Walmajarri, Wangaaybuwan-Ngiyambaa, Wangganguru, Wangka-Wiru, Wargamay, Warlpiri, Warluwara, Warrnambool, Warungu, Wathawurrung, Wembawemba, Wemba-Wemba, Westernarrernte, Wik-Mungkan, Wiradhuri, Wirangu, Wirri, Woiwurrung, Worimi, Wulguru, Wuliwuli, Yalarnnga, Yanango, Yandruwandha,

Yanyuwa, Yaygir, Yidiny, Yindjibarndi, Yir-Yoront, Yitha-Yitha, Yolngumatha, Yorta-Yorta, Yugambal, Yulparija, Rainbarngo, Ngangikurrunggurr, Ngengomeri, Ganggalida, Kayardild, Tiwi, Umbugarla, Wagiman, Waray, Djingili, Gudanji, Wambaya, Ami, Manda, Maramanadji, Maranunggu, Marengar, Maridan, Marithiel, Marityaben, Marriammu, Gunin-Kwini, Ngarinyin, Unggumi, Wunambal, Wardaman, Yangman

2. **Population:** Central Asia (CA)

- **Language family**: Altaic; Sino-Tibetan

- **Candidate doculects:**
    - 3 Altaic doculects: Uyghur, Kalmyk, Uzbek
    - 3 Sino-Tibetan doculects: Kunming Mandarin, Mandarin, Mandarin2

3. **Population:** East Africa (EA)

- **Language family:** Afro-Asiatic; Nilo-Saharan

- **Candidate doculects:**
    - 5 Afro-Asiatic doculects: Amharic, Banna, Walani Silte, Tigrinya, Afar
    - 2 Nilo-Saharan doculects: Turkana, Nyangatom

4. **Population:** Japan (JP)

- **Language family**: Japanese

- **Candidate doculects**: (14 doculects)
  Japanese, Japanese 2, Japanese Kyoto, Kamikatetsu Kikai, Miyako, Naha, Northern Amami Oshima, Shuri, Tokuwase, Tokyo Japanese, Yaeyama, Yonaguni, Yonamine, Yoro

5. **Population:** Melanesia (ME)

- **Language family:** Austronesian; Savosavo; Solomons East Papuan; Touo

- **Candidate doculects**:

    - 205 Austronesian doculects:
      Kahua, Tawaroga Kahua, Ax¬Amb, Axamb Maxbaxo, Axamb Avok, Arosi Tawatana, Oneibia Arosi, Araki, Amblong, Marau, Maasupa Areare, Waiahaa Areare, Aore, Ulawa Saa, Uki Ni Masi Saa, Aulu Saa, Saa, Suru Kavian Apma, Apma, Aneityum 2, Asumboa, Anuta, Aulua, Avaso Babatana, Sengga Babatana, Sisiqa, Tunoe Babatana, Lomaumbi Babatana, Katazi Babatana, Dhad¬Haje Boghotu, Bughotu, Baki, Blablanga, Ghove Blablanga, Bierebo Bonkovia, Bierebo Yevali, Bierebo Tavio, Bierebo Burupika, Butmas, Tur,

Dakaka Baiap, Dakaka Sesivi, Bieria Vovo, Mbirao, Bae¬Tora Navenevene, Baetora Tam, Baetora Nasawa, Baetora Narovorovo, Baetora, Mbaelelea, Mbaengguu, Baroo Bauro, Haununu Bauro, Bauro, Rawo, Dixon Reef 2, Dixon Reef 1, Dorio, Sie, South Efate Erakor, South Efate Pango, South Efate Eratap, Eton Vanuatu, Fagani, Fa¬Taleka, Fortsenal, Aniwa, Futuna, Kiribati, Kwai, Nggeri Ghari, Ndi Ghari, Nggae Ghari, Nginia Ghari, Tandai Ghari, Ghari, Hiw, Hoava, Zazao, Zabana, Kokota, Koro, Kusaghe, Kwaio, Kwaraae, Kazukuru, Lungga, Langalanga, Lengo, Lengo 2, Longgu, Lakona, North Efate Siviri, North Efate Nguna, North Efate Sesake, North Efate Pwele, North Efate Woraviu, Walade Lau, Lau North, Lau, Raga, Lamenu, Malango, Toabaita, North Ambrym Ranon, North Ambrym Fonah, Emae, Malmariv, Rennellese, Mbareke, Cheke Holo, Leleghia Maringe Cheke Holo, Tataba Maringe Cheke Holo, Kmagha Maringe Cheke Holo, Mosina, Mosina Vetumboso, Alu Mono, Fauro Mono, Mono Alu, Mono, Marovo, Nanggu, Duke, Nggela, Nam¬Bakaengo Malo, Banua, Nea, Big Nambas Unmet, Big Nambas Leviamp, West Ambae, Nea Nooli, Nea Nemboi, Southwest Tanna Ikiyau, South¬West Tanna Ikiti, Southwest Tanna Imreang, Southwest Tanna Lapwangtoai, Southwest Tanna Enfitana, Luangiua, East Ambae Wailengi, East Ambae Lolsiwoi, East Ambae Lolomatui, Unua, Oroha, Vaeakau Taumako, Pileni, Ririo, Roviana, Simbo, Ske, Seke, Sikaiana, Shark Bay 2, Shark Bay 1, Santa Ana Owa, Sowa, Tan¬Goa, Tikopia, Teanu, Tolomako, Tolo, Malagheti Talise, Koo Talise, Poleo Talise, Moli Talise, Tambotalo, Katbol, Katbol Timbembe, Tu¬Tuba, Tasmate, Kwamera Yatukwey, Kwamera Isiai, Kwamera Port Resolution, North Tanna, Whitesands Iarkei, White¬Sands Loniel, Tanema, Vaghua, Southeast Ambrym Maat, Southeast Ambrym Toak, Ughele, Uri, Rano, Atchin, Tautu, Wala, Pinalum, Uripiv, Ura, Vao, Valpei Hukua, Valpei, Vano, Vunapu, Vatrata, Varisi, Burmbar, Burmbar Vartavo, Burm¬Bar Lepaxsivir, Wailapa, Wusi Kerepua, Wusi Valui, Wusi Mana, Wusi Nonona, Wetamut Dorig, Wetamut

- 1 Savosavo doculect: Savosavo
- 3 Solomons East Papuan doculects: Bilua, Ndovele Bilua, Lavukaleve
- 1 Touo doculect: Mbaniata

6. **Population:** New Caledonia (NC)

- **Language Family:** Austronesian

- **Candidate doculects**: (16 doculects)
  Xaracuu, Cemuhi, Grand Couli, Dehu, Dumbea, East Futuna, Futuna East, Iaai, Jawe, Fa Tieta, Nelemwa, Nemi, Nengone, West Uvean, Wallisian, Wallisian East Uvean

7. **Population:** Philippines Aeta/Agta “Negrito” (NE)

- **Language Family:** Austronesian

- **Candidate doculects:** Agta, Dupaningan Agta, Umiray Dumaget Agta

8. **Population:** Papua New Guinea (NG)

- **Language Family:** Austronesian; Anêm; Dagan; Eleman; Inland Gulf; Kiwaian; Kwalean; Morehead and Upper Maro Rivers; Trans-New Guinea; Teberan-Pawaianl Turama-Kikorian; Western Fly; Yareba; Other (Pidgins and Creoles)

- **Candidate doculects:**

  - 104 Austronesian doculects:
    Adzera, Aiklep, Amara, Amari, Arawe, Are, Aribwatsa, Arop Lokep, Azera, Bariai, Barim, Bilbil, Biliau, Buang, Buang Mapos, Buasi, Bubwaf, Bugawac, Buhutu, Bukaua, Bunama, Bwaidoka, Dagin, Dambi, Dangal, Diodio, Dobu, Doura, Dunguntung, Duwet, Gabadi, Gapapaiwa, Gitua, Gumawana, Guwot, Hote, Iamalele, Jabem, Kaiwa, Kaulong, Keapara, Kela, Kilivila, Kumaru, Kuni, Labu, Lala, Lamogai, Latep, Lukep, Magori, Maisin, Malasanga, Maleu, Maleu 2, Manga, Mangap, Mapos, Maralango, Mari, Mato, Mbula Papua New Guinea, Mekeo, Minaveha, Molima, Motu, Mouk 1, Mouk 2, Musom, Musom 2, Mutu, Nengaya, Numbami, Onank, Patep, Piu, Roinji, Roro, Saliba Papua Newguinea, Sambio, Sengseng, Sewa Bay, Silisili, Sinaugoro, Sio, Sipoma, Sirak, Sirasira, Suau, Sukurum, Tami, Tawala, Towangara, Tuam, Ubir, Wagau, Wampar, Wampar 2, Wampur, Wedau, Yabem, Yamap, Yaros, Zenag
  - 1 Anêm doculect: Anêm
  - 1 Dagan doculect: Dagan
  - 19 Eleman doculects: Aheave, Kaipi, Karaeta Uaripi, Keuru, Luluitera Uaripi, Meii2 Uaripi, Murua Stmt Uaripi, Opao, Petoe Uaripi, Orokolo, Orokolo 2, Sepoe, Siviri Uaripi, Toaripi, Toaripi 2, Uaripi, Uaripi Uaripi, Purari, Kaki Ae
  - 2 Inland Gulf doculects: Ipiko, Minanibai
  - 11 Kiwaian doculects: Anigibi, Bamu, Bamu 2, Gibaio, Gope, Kerewo, Kibiri, Kiwai Southern, Morigi, Urama, Wabuda
  - 6 Kwalean doculects: Humene Manugoro, Humene, Kwale, Mulaha Iaibu, Mulaha Mulaha, Mulaha
  - 1 Morehead and Upper Maro Rivers doculect: Tabo Waia
  - 139 Trans-New Guinea doculects:
    Ampale, Angaataha, Ankave, Ankave 2, Baruya, Baruya 2, Hamtai, Ivori, Kamasa, Kapau, Kawacha, Lohiki, Menya, Menya 2, Simbari, Tainae, Yagwoia, Binandere, Korafe Yegha, Mambare River, Suena, Tafota Baruga, Zia, Boumai, Dom, Golin, Kuman, Middle Wahgi, Sinasina, Agaribi, Alekano, Asaro, Auyana, Awa, Awa 2, Benabena, Binumarien, Fore, Gadsup Agarabi, Gadsup, Gafuku, Gahuku Asaro, Gahuku, Gende, Gimi, Isabi, Kamano Kafe, N Tairora, Siane, Tairora Binumarien, Waffa, Yabiyufa, Yagaria, Yate, Kewa, Kewa S Pole, Pole, Sau, Awara, Borong, Burum, Burum Mindik, Dedua, Hube, Kate, Komba, Kosorong, Mape, Mape 2, Migabac, Mindik, Momolili, Nabak, Nankina, Nek, Nukna, Ono, Selepet, Timbe, Tobo, Wantoat, Yopno, Afoa, Kunimaipa, Mafulu, Aomie, Barai, Ese Managalasi, Koiari, Koiari 2, Koita, Mountain Koiari, Mountain Koiari 2, Arawum, Asas, Bau, Biyom, Bom, Bongu, Danaru, Duduela, Dumpu, Erima, Faita, Ganglau, Girawa, Jilim, Kesawai, Kolom, Kwato, Lemio, Male Papuang, Pulabu, Rerau, Saep, Sausi, Sihan, Sinsauru, Songum, Sumau, Suroi, Tauya, Urigina, Usino, Usu, Yabong, Yangulam, Domu, Laua, Mailu, Mailu 2, Doromu Aramaika, Doromu Bareika, Doromu Lofaika, Doromu, Maria Maranomu 1, Maria, Makayam, Wiru
  - 3 Teberan-Pawaian doculects: Pawaia, Daribi, Folopa

- 3 Turama-Kikorian doculects: Mena, Omati, Rumu
- 18 Western Fly doculects: Bine Kunini, Gizrra Kupere, Gizrra Togo, Gizrra Waidoro, Meriam, Ume, Wipi Abam, Wipi Dorogori, Wipi Gamaewe, Wipi Guiam, Wipi Iamega, Wipi Kapal, Wipi Kuru, Wipi Peawa, Wipi Podari, Wipi Wipim, Wipi Wonie, Wipi Yuta
- 1 Yareban doculect: Yareba
- Other (Pidgins and Creoles) doculects: Motu Hiri, Tok Pisin, Torres Strait Creole

9. **Population:** North India (NI)

- **Language Family:** Indo-European
- **Candidate doculects:** Bengali

10. **Population:** South Africa (SA)

- **Language Family:** Khoisan; Niger-Congo
- **Candidate doculects:**
  - 2 Khoisan doculects: Khoekhoegowab, Nama
  - 12 Niger-Congo doculects: Xhosa 2, Xhosa, S41 Xhosa, North¬Ern Sotho, Sotho Northern, Sotho Southern, Sotho Sud, Zulu 2, Zulu Nkandla, Zulu, Tswana 2, Tswana

11. **Population:** South India (SI)

- **Language family:** Dravidian
- **Candidate doculects:** Tamil
